# Characterization and genomic analysis of two novel psychrotolerant *Acidithiobacillus ferrooxidans* strains from polar and subpolar environments

**DOI:** 10.3389/fmicb.2022.960324

**Published:** 2022-08-24

**Authors:** Claudia Muñoz-Villagrán, Jonnathan Grossolli-Gálvez, Javiera Acevedo-Arbunic, Ximena Valenzuela, Alonso Ferrer, Beatriz Díez, Gloria Levicán

**Affiliations:** ^1^Departamento de Biología, Facultad de Química y Biología, Universidad de Santiago de Chile (USACH), Santiago, Chile; ^2^Programa de Biorremediación, Campus Patagonia, Universidad Austral de Chile, Valdivia, Chile; ^3^Núcleo de Química y Bioquímica, Facultad de Ciencias, Ingeniería y Tecnología, Universidad Mayor, Santiago, Chile; ^4^Departamento de Genética Molecular y Microbiología, Pontificia Universidad Católica de Chile, Santiago, Chile; ^5^Center for Climate and Resilience Research (CR)2, Santiago, Chile; ^6^Center for Genome Regulation (CRG), Santiago, Chile

**Keywords:** *Acidithiobacillus*, iron-oxidizing bacteria, cold adaptations, acidophiles, Antarctic, Chilean Patagonia

## Abstract

The bioleaching process is carried out by aerobic acidophilic iron-oxidizing bacteria that are mainly mesophilic or moderately thermophilic. However, many mining sites are located in areas where the mean temperature is lower than the optimal growth temperature of these microorganisms. In this work, we report the obtaining and characterization of two psychrotolerant bioleaching bacterial strains from low-temperature sites that included an abandoned mine site in Chilean Patagonia (PG05) and an acid rock drainage in Marian Cove, King George Island in Antarctic (MC2.2). The PG05 and MC2.2 strains showed significant iron-oxidation activity and grew optimally at 20°C. Genome sequence analyses showed chromosomes of 2.76 and 2.84 Mbp for PG05 and MC2.2, respectively, and an average nucleotide identity estimation indicated that both strains clustered with the acidophilic iron-oxidizing bacterium *Acidithiobacillus ferrooxidans*. The Patagonian PG05 strain had a high content of genes coding for tolerance to metals such as lead, zinc, and copper. Concordantly, electron microscopy revealed the intracellular presence of polyphosphate-like granules, likely involved in tolerance to metals and other stress conditions. The Antarctic MC2.2 strain showed a high dosage of genes for mercury resistance and low temperature adaptation. This report of cold-adapted cultures of the *At. ferrooxidans* species opens novel perspectives to satisfy the current challenges of the metal bioleaching industry.

## Introduction

The genus *Acidithiobacillus* comprises a number of Gram-negative acidophilic bacteria that thrive in acidic environments, such as volcanic areas including acidic ponds, lakes and rivers, sulfur springs, and acid mine/rock drainages (AMD/ARD) around the world (Núñez et al., 2017). This genus consists of a group of rod-shaped bacteria that derive energy from the oxidation of elemental sulfur and reduced sulfur compounds to support autotrophic growth by using the Calvin-Bassam-Benson cycle (Wang et al., 2019). The species *At. ferrooxidans*, *At. ferrivorans, At. ferridurans*, *At. ferriphilus*, and *At. ferrianus* can also catalyze the dissimilatory oxidation of ferrous iron (Fe^2+^; Amouric et al., 2011; Hedrich and Johnson, 2013; Falagán and Johnson, 2016; Norris et al., 2020). Because of their metabolic capabilities, the *Acidithiobacillus* spp. are important players in the biogeochemical cycle of iron and sulfur in a variety of natural and anthropogenic environments.

Members of the *Acidithiobacillus* genus and other iron-oxidizing microorganisms such as *Leptospirillum*, *Acidiphilum*, *Acidiferrobacter*, *Ferrovum*, *Alicyclobacillus*, *Ferrimicrobium*, *Acidimicrobium* and *Ferrithrix* are also relevant in the industrial bioleaching of sulfide minerals to recover copper and other valuable metals (Johnson and Hallberg, 2005; Schippers et al., 2014; Shiers et al., 2016). The use of these bacteria for the processing of electronic and electric wastes, and slags, and desulfurization of coal has also been explored as a very promising alternative for re-cycling and generation of raw materials (Yang et al., 2014, 2015; Priya and Hait, 2017; Auerbach et al., 2019). The bioleaching process occurs through the oxidation of Fe^2+^ present in sulfide minerals such as pyrite (FeS_2_) and chalcopyrite (CuFeS_2_). This process allows the bacteria to synthesize ATP for supporting cellular function and growth, and simultaneously leads to the regeneration of ferric iron (Fe^3+^), which conducts the chemical leaching of the minerals, thus facilitating metal solubilization (Vera et al., 2013). Biomining is thus a well-established practice characterized by requiring a simpler infrastructure as no contaminating sulfur dioxide is produced compared to traditional pyrometallurgical processes (Shiers et al., 2016).

The bioleaching of low-grade metal sulfides is often carried out in heaps of crushed or run-of-mine ores where biomining microorganisms are exposed to very harsh conditions that include high osmotic potential due to the accumulation of ions in recircularized water, a high load of toxic metals such as iron and copper, and changes in temperature or extreme temperatures determined by the climatic conditions where the industrial operation is taking place (Shiers et al., 2016). In cold environments, like those found at low and high latitudes and at high altitudes, low temperature becomes a limiting factor (Dopson et al., 2007). In Chile currently, there are a number of mining companies located in the Andes Mountains, where the temperature remains near or below 0°C for 6 months or more in the year (Escobar et al., 2010; Gentina and Acevedo, 2013).

While most species in the genus *Acidithiobacillus* are mesophilic microorganisms, a smaller number also include moderately thermophilic ones (*At. albertensis* and *At. caldus*). Interestingly, *At. ferrivorans* is an iron oxidizing eurypsychrophile that has a wide temperature tolerance (Christel et al., 2016). This bacterium has been identified and/or isolated from various cold sites worldwide including Russia and the Altiplano in South America (Hallberg et al., 2010; Liljeqvist et al., 2012; Barahona et al., 2014; Talla et al., 2014; Ccorahua-Santo et al., 2017). Of note is that this microorganism was also detected in Arctic and Antarctic iron-rich outflows (Blood Falls) through the use of molecular tools (Dold et al., 2013; García-Moyano et al., 2015). Other psychrotolerant iron oxidizers include *Ferrovum myxofaciens* that was detected in a permanently cold AMD system (Johnson et al., 2014) and *At. ferriphilus* (Falagán and Johnson, 2016), which can grow even at 5°C. In addition, the presence and leaching activity of iron and sulfur-oxidizing bacteria has been detected in ore materials and enrichment cultures obtained from low-temperature sites (Ahonen and Tuovinen, 1992; Langdahl and Ingvorsen, 1997; Elberling et al., 2000), opening the possibility that other psychrotolerant acidophiles may yet be recovered and characterized.

Mechanisms to tolerate growth at low temperature have been predicted in *At. ferrivorans* (Rudolph et al., 1986; Dopson et al., 2007; Liljeqvist et al., 2012; Christel et al., 2016). However, given the combination of extreme environments that arise in the industrial operation of leaching, the microorganisms must be able to tolerate and be active under a variety of conditions that also require metal and osmotic tolerance. In this way, the search for new isolates of iron-oxidizing bacteria with the required properties has gained increasing attention in the last decade as a potential alternative technology, and it is clear that more efforts have to be focused toward the isolation and characterization of these microorganisms.

In this work, we obtained, characterized and identified iron-oxidizing bacteria from Chilean Patagonia and Antarctic. Two cultures were characterized as *At. ferrooxidans*, and both have the ability to grow in iron, at an optimal temperature of 20°C. The ultrastructure studies highlighted the presence of intracellular polyphosphate-like deposits. The analysis of the genome sequences of these bacteria revealed interesting features concerning potential mechanisms for adaptation to metal and low temperature.

## Materials and methods

### Site description and sample collection

**FIGURE 1:**
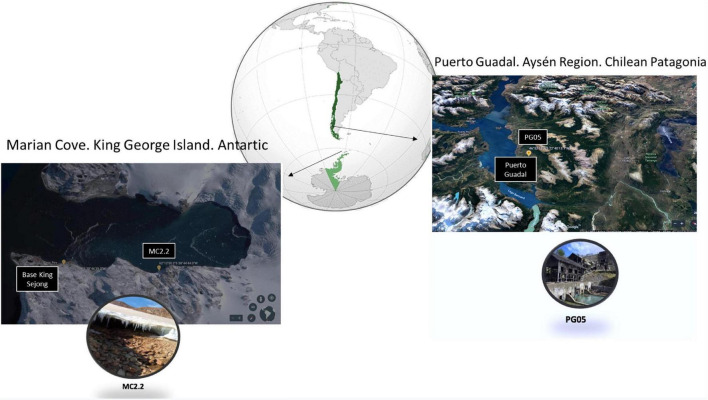
Location of the sample collection sites. The insets are close-up views of AMDs in Marian Cove **(left)** and the Escondida mine in Puerto Guadal **(right)**. Map from Google Earth.

Three soil and superficial sediment samples per sampling site were collected in an abandoned lead/copper mine in Puerto Guadal in Aysén Region, Chile (46°52′13.6″S 72°40′13.1″W) and Marian Cove, King George Island, Antarctic (62°12 ′S 58°44 ′W; Figure 1), and stored in sterile flasks at 4°C until further analysis. The physicochemical analysis was undertaken using 1 g sediment sample suspended in 20 mL distilled water; afterward, the pH, salinity, oxidation reduction potential (ORP) and conductivity was measured by a multiparameter Hannah HI9828 equipment.

Soil samples were analyzed for Fe, S, Cu, Pb and Zn content in a Spectro CIROS Vision ICP-OES instrument using the wavelengths of 238.204, 181.975, 327.393, 220.353, and 206.200 nm, respectively. A calibration curve was constructed using commercially available metal standards (Merck) in 10% HNO_3_.

### Enrichment cultures and growth conditions

Sediment samples were also used to inoculate in culture media to 1% w/v, and were grown aerobically in shake flasks at 20°C and constant stirring at 180 rpm. The media used were 9K [0.04 g/L K_2_HPO_4_; 0.01 g/L MgSO_4_*7H_2_O; 0.1 g/L (NH_4_)_2_SO_4_; 25.76 g/L FeSO_4_*7H_2_O; pH 1.6] (Rivera-Araya et al., 2020); 9KM1 [0.5 g/L K_2_HPO_4_; 0.5 g/L MgSO_4_*7H_2_O; 3 g/L (NH_4_)_2_SO_4_; 0.1 g/L KCl; 0.01 Ca(NO_3_)_2_*4H_2_O; 2 g/L MnSO_4_; 2.1 g/L Al_2_(SO_4_)*18H_2_O; 48 g/L FeSO_4_*7H_2_O; pH 1.6] (Liu et al., 2007); 9KM2 [0.5 g/L K_2_HPO_4_; 0.5 g/L MgSO_4_* 7H_2_O; 3 g/L (NH_4_)_2_SO_4_; 0.1 g/L KCl; 0.01 Ca(NO_3_)_2_*4H_2_O; 45.22 g/L FeSO_4_*7H_2_O; pH 3.3] (Dixit et al., 2018); and DSMZ 882 [0.132 g/L (NH_4_)_2_SO_4_; 0.053 g/L MgCl_2_*6H_2_0; 0.027 g/L KH_2_PO_4_; 0.147 g/L CaCl_2_*2H_2_O; 18.40 g/L FeSO_4_*7H_2_O; pH 1.8]. The growth of iron-oxidizing cultures was followed by direct counting under an optical microscope with a Neubaüer-Improved chamber (0.02 mm, depth), and by the appearance of a reddish coloration of the medium due to the oxidation of Fe^2+^ to Fe^3+^.

### Determination of optimal growth temperature

In order to determine the optimum growth temperature of the bacterial enrichment, 150 mL of fresh 9KM1 medium were inoculated at 10% v/v with enriched iron-oxidizing cultures. The cultures were incubated at 5, 20, 30 and 37°C with constant shaking at 180 rpm. Each culture was adapted by two successive culturing at each temperature. Cell growth was evaluated until the stationary phase of growth was reached, or after 14 days. Bacterial growth curves were plotted using the GraphPad Prism 8 version 8.0.2 program. For calculation of specific growth rate (μ), we selected cell densities recorded at the exponential phase of each strain, and we calculated the specific growth rate using the following equation:


k=(ln⁢(N2/N1))/(t2-t1)

Where k is the specific rate, N1 and N2 are the initial and terminal cell density recorded, respectively, and t1 and t2 correspond to initial and terminal time, respectively (in hours). Thus, the unit of *k* is h-1. The generation time (or doubling time) was calculated according to the following equation:


G⁢e⁢n⁢e⁢r⁢a⁢t⁢i⁢o⁢n⁢t⁢i⁢m⁢e:(ln⁢(2))/k

### Electron microscopy

Cell morphology and size were examined by scanning electron microscopy (SEM, Hitachi) and transmission electron microscopy (TEM, Tecnai), respectively. Cell cultures in 250 mL of 9KM1 were incubated at 20°C with constant shaking at 180 rpm until they reached the late exponential growth phase. Then, the cells were collected by centrifugation and washed twice with 1 mL of 10 mM sodium citrate pH 7.0. Finally, the cells were fixed with 50% v/v glutaraldehyde solution and sent to the Advanced Microscopy Unit (UMA) of the Pontifical Catholic University (PUC), Santiago, Chile, for further treatment and analysis.

### Deoxyribonucleic acid extraction

The cells were collected from 1 L culture by centrifugation at 8000 × *g* for 30 min at 4°C and washed twice with 50 mL of 10 mM sodium citrate pH 7.0, to eliminate the remnant iron. The total DNA from each pellet was obtained with a commercial Wizard^®^ Genomic DNA Purification Kit (Promega) following the fabricant’s indications. The concentration and purity of the genomic DNA was determined with a Nanodrop Take3 spectrophotometer (Biotek).

### Deoxyribonucleic acid sequencing and annotation

16S rDNA from enrichment culture was amplified by using the bacterial universal primer set 27F 5′-GGGGTTTGATCCTGGCTCAG-3′ and 1387R 5′-GGGCGGNGTGTACAAGGC-3′ (Marchesi et al., 1998). The PCR products were sent to the Sequencing Unit (UMA) of the Pontifical Catholic University (PUC), Santiago, Chile. Obtained sequences were analyzed and compared using SnapGene 6.0.2, and Blast tool from National Center for Biotechnology Information (NCBI^1^).

The draft genome sequences of *At. ferrooxidans* MC2.2 and PG05 strains were determined by a whole-genome shotgun strategy using a pair-end library in the Illumina MiSeq platform (Andes Genomics). A total of 4 million reads were quality filtered and assembled in SPAdes v. 3.13.0 under default parameters (Nurk et al., 2013). Assembly annotation was carried out using the NCBI Prokaryotic Genome Annotation Pipeline (PGAP), as described.^2^ The circular genome map was assembled using plotMyGBK wrapper script^3^; plotMyGBK uses BioPython and the R platform with the packages rSamTools, OmicCircos, and data.table to produce a vector image of a circular map (Morgan et al., 2016^4^). The sequence assembly accession numbers at NCBI database are GCF_017165965.1 and SAMN17216810 for PG05 and MC2.2 strains, respectively.

### Phylogenetic relationships and whole-genome nucleotide identity

For phylogenetic tree construction, thirty phylogenetic marker genes corresponding to widespread housekeeping genes *dnaG, nusA, rplA, rplD, rplK, rplN, rpsB, rpsI, rpsM, tsf, frr, pgk, rplB, rplE, rplL, rplP, rpmA, rpsC, rpsJ, rpsS, infC, pyrG, rplC, rplF, rplM, rplS, rpoB, rpsE, rpsK*, and *smpB* were identified in *Acidithiobacillus* strains PG05 and MC2.2 using AMPHORA2 (Wu and Eisen, 2008; Mende et al., 2013). Each gene was translated under standard genetic code to perform a protein-coding-guided multiple nucleotide sequence alignment, using TranslatorX MUSCLE for the multiple sequence alignment (Abascal et al., 2010). Alignments were concatenated using the alignment editor tool Seqotron (Fourment and Holmes, 2016) and the best partition scheme and substitution model was evaluated by PartitionFinder 2 (Lanfear et al., 2017). Finally, the software MrBayes v3.2 was used for phylogenetic reconstruction (Ronquist et al., 2012), and the resulting tree was plotted and annotated using FigTree v1.4.3.^5^ For the phylogenetic tree, Maximum Likelihood was the best fitting model for these sequence data. For analysis, the genome sequences from thirty-one *Acidithiobacillus* strains were collected in Supplementary Table 1 (Supporting information). The average nucleotide identity (ANI) was calculated for the 31-genome dataset using the pyani Python3 module (Pritchard et al., 2016) and the results were visualized using the data.table and pheatmap R packages.

### *At. ferrooxidans* pangenome analysis

The analysis was carried out following the anvi’o pangenomic workflow (Delmont and Eren, 2018). Functional annotation of genes was performed using HMMER (Wheeler and Eddy, 2013) and COGs (Tatusov et al., 2001). Genome storages were generated using “anvi-pan-genome” with parameters “–minbit 0.5″ to remove weak hits (Benedict et al., 2014) and “–mcl-inflation 10″ to identify gene clusters (Van Dongen and Abreu-Goodger, 2012). The ANI in genomes of the *At. ferrooxidans* strains was computed using pyANI (Pritchard et al., 2016). Finally, “anvi-display-pan” was used to visualize the distribution of gene clusters across genomes.

Additionally, using the temperature variables an analysis of enriched functions was performed comparing the mesophilic versus the psychrotolerant strains (“anvi-get-enriched-functions-per-pan-group”). To analyze the global functional differences between the full and accessory genes, the genomes of PG05, MC2.2, and ATCC 27230 strains were annotated with eggNOG-mapper (Huerta-Cepas et al., 2017) and their COG composition visualized with ggplot2 R package (Wickham, 2016).

### Searching for genes for environmental resistance

Amino acid sequences derived from genes identified as being involved in the stress responses were used as query sequences to search the translated nucleotide database from the genomes of the *At. ferrooxidans* PG05 (DSM 102806) and *At. ferrooxidans* MC2.2 (JCM5614) strains using tBlastn (Gertz et al., 2006) with default parameters. When a prospective candidate gene was identified, its predicted amino acid sequence was used to perform a BlastP (Pearson, 2013) search of the NCBI non-redundant database. Only the best hits were accepted as evidence for putative orthologs.

### Statistical analysis

Statistical analysis was performed using the one-way ANOVA test followed by Tukey’s, using GraphPad Prism 5. The differences were considered to be significant at *P* < 0.05.

## Results and discussion

### Physicochemical analysis of samples and obtaining of iron-oxidizing microbial enrichments

Physicochemical parameters (pH, salinity, redox potential, and conductivity) were determined in the sediment samples collected in Patagonia (February 2019) and in Antarctic (December 2018), as shown in Table 1 and Figure 1. The value range for each parameter fluctuated importantly between the different samples. It is notable that the recorded pH values varied between 3.6 and 6.7. The samples from the abandoned mine in Patagonia (PG samples) were in general at acidic pH, suggesting the presence and activity of iron- and sulfur-oxidizing microorganisms (Schippers et al., 2014).

**TABLE 1:** The physicochemical parameters of sediment samples collected at Marian (MC) and Puerto Guadal (PG).

Sample	pH	Salinity (PSU)	ORP (mV)	Conductivity (μS/cm)
PG01	3.6	0.12	343.0	248
PG03	4.6	0.03	293.0	62
PG05	4.2	0.04	325.0	91
MC1.1	5.2	0.22	115.5	462
MC1.2	5.8	0.06	35.3	134
MC2.1	4.8	0.02	66.8	47
MC2.2	5.7	0.02	47.4	55
MC3.2	5.4	0.03	121.7	65
MC5.1	6.1	0.11	88.1	226
MC6.1	5.8	0.23	87.3	465
MC6.2	5.8	0.29	94.8	591
MC8.2	6.7	0.06	19.9	136
MC8.3	6.2	0.07	43.7	146
MC10.2	5.6	0.01	100.4	30
MC12	5.6	0.02	93.6	51
MC13	5.7	0.02	88.0	53

For microbial enrichment, 9K, 9KM1, 9KM2, and 882 culture media containing ferrous sulfate as an electron donor and acidic pH, were used to favor the thriving of acidophilic iron-oxidizers as described in Materials and Methods. As shown in Table 2, in fourteen out of sixteen samples, microbial growth and iron oxidation were detected. Samples PG05 (Patagonia) and MC2.2 (Antarctica) were selected due to their higher microbial density and iron oxidation capacity, as evidenced by the generation of a reddish color in the culture media (Johnson et al., 2012). It is noteworthy that microbial enrichments derived from two samples were able to grow in 9KM1 and 9KM2 culture media, which have usually been used for *Acidithiobacillus* culturing (Liu et al., 2007; Dixit et al., 2018). Finally, it is important to remark that both enrichments were able to grow in 9K medium at pH 3.5 when the ferrous iron (Fe_2_SO_4_) was replaced by elemental sulfur as the only energy source (data not shown). The ability to grow in this condition was verified by the increase in cell density. Thus, according to our results, it can be stablished that PG05 and MC2.2 cultures can oxidize both ferrous iron and elemental sulfur as primary energy sources.

**TABLE 2:** Evaluation of the bacterial growth of the samples in the different acid culture media.

Sample	9K	9KM1	9KM2	882
PG01	X	X	X	X
PG03	X	X	X	X
PG05	x	***	**	X
MC1.1	–	*	–	–
MC1.2	*	*	*	**
MC2.1	–	*	*	X
MC2.2	x	***	***	X
MC3.2	–	*	–	–
MC5.1	–	*	–	–
MC6.1	–	**	–	–
MC6.2	–	**	**	–
MC8.2	–	**	–	–
MC8.3	–	**	–	–
MC10.2	–	*	–	–
MC12	**	***	**	**
MC13	–	*	–	–

*Low growth; **Medium growth; ***High growth; –Not evaluated; X Without growth.

Common traits of the sediment samples containing PG05 and MC2.2 are their low salinity (0.02–0.04 PSU) and conductivity (47–91 μS/cm), while in other samples these variables can reach values as high as 0.22 PSU and 462 μS/cm, respectively. The samples PG05 and MC2.2 contained iron (779/597 mg/L) and sulfur (149.56/59.3 mg/L) (Table 3). PG05 samples also harbored significant amounts of copper (21.6 mg/L), lead (24.9 mg/L) and zinc (3.0 mg/L) which is in agreement with the previous mining activity of these metals in the disused mine from where PG samples were collected. These data suggest that the PG05 strain has the ability to tolerate high concentrations of dissolved metals. The presence of iron and sulfur in the PG and MC samples certainly supports the presence of microorganisms able to transform these elements. Iron was previously detected and measured in Antarctic locations including the Marian Cove area (Dold et al., 2013). Through a metagenomic study, these researchers described microbial diversity and proposed the biogeochemical processes related to acid rock drainage generation, and rock erosion in the Antarctic landmass. An indirect indication of pyrite oxidation in the Antarctic was reported by the observation of schwertmannite present in glacier ice and icebergs (Raiswell et al., 2009), and extensive sulfide mineralization (Hawkes, 1982). Additionally, sulfide oxidation liberates and mobilizes iron, sulfur, and heavy metals, bringing them to an aqueous phase. This process results in the formation of a Fe^2+^-plume, which flows toward the sea as dissolved iron in the form of groundwater in the Marian Cove area (Dold et al., 2013). Unlike the expedition carried out by Dold et al., we were not able to observe the described plume; however, the content of iron that we detected suggests that an intensive biological activity could be participating in the biogeochemical cycle of this element.

**TABLE 3:** Determination of the concentration of metals in sediment samples using ICP-EOS.

Sample	Metal concentration (mg/L)				
	
	Fe	S	Cu	Pb	Zn
PG05	779	59.3	21.6	24.9	3.0
MC2.2	597	149.5	0	0	0.4

### Identification and microscopic characterization of PG05 and MC2.2 cultures

**FIGURE 2:**
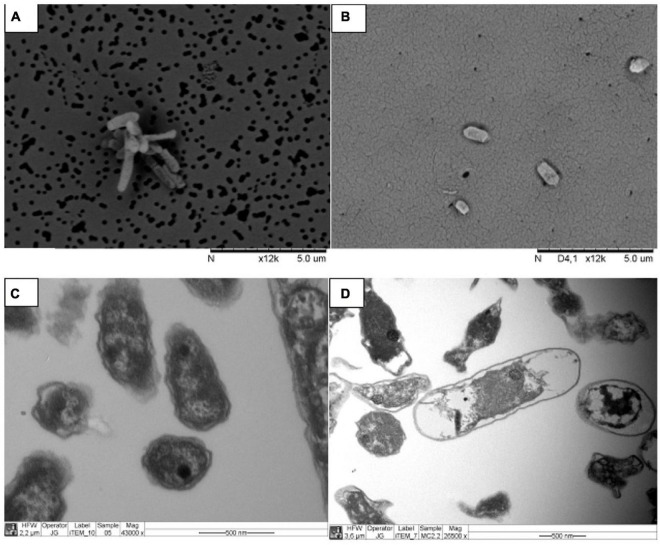
Electron microscopy images of strains grown at 20 °C. Strains PG05 **(A)** and MC2.2 **(B)** visualized by SEM. Strains PG05 **(C)** and MC2.2 **(D)** visualized by TEM.

Enrichment cultures were subcultured three times in liquid 9KM1 medium before the molecular identification. The 16S DNA sequence analysis of PG05 (MW458966) and MC2.2 (MN985509.1) cultures revealed the presence of one unique (or dominant) phylotype in each culture with similarities of 100% to *Acidithiobacillus ferrooxidans*. Further, the strains were characterized by electron microscopy. Analysis by SEM of cells harvested at the late exponential phase revealed that the PG05 strain has a rod-shaped cellular form that is approximately 2 μm long and 0.45 μm in diameter (Figure 2A), and MC2.2 strain has the rod-shaped with an average size of 1.5 μm long and 0.45 μm in diameter (Figure 2B). The size and shape observed agree with those described for other representatives of the *Acidithiobacillus* genus (Kelly and Wood, 2000).

From the TEM images it is also possible to observe electron-dense areas inside the cells, a trait that is especially prominent in the PG05 strain (Figures 2C,D). The presence of electron-dense zones has been reported to occur due to the presence of polyphosphate granules (polyP), a polymer of phosphoanhydride-linked phosphate residues, found as chains up to 1,000 residues long in cells (Jiménez et al., 2017). This biopolymer plays a relevant role in the response of bacteria to stress conditions including heat, starvation, acid, and oxidative stress (Gray and Jakob, 2015; Kumar et al., 2016; Tiwari et al., 2019). In acidophiles, polyP has been identified in *At. ferrooxidans, At. ferrivorans*, and *At. thiooxidans* where they have been shown to be involved in tolerance to metals (Álvarez and Jerez, 2004; Navarro et al., 2013; Ulloa et al., 2018). Thus, this intracellular biopolymer may have an important role in tolerance and fitness of the PG05 strain.

### Optimal growth temperature of the PG05 and MC2.2 cultures

**FIGURE 3:**
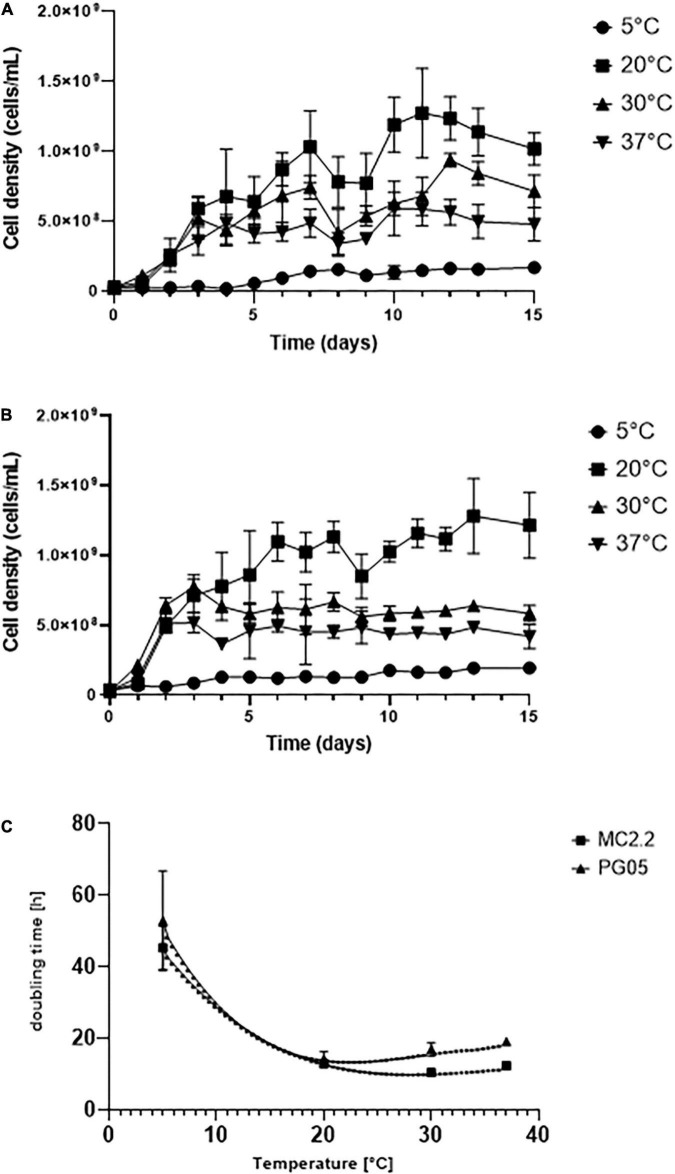
Growth of *Acidithiobacillus* strains incubated at different temperatures. **(A)** PG05. **(B)** MC2.2. **(C)** Effect of the temperature on doubling times of the strains.

In order to determine the optimum growth temperature of the cultures, growth curves of each strain supplemented with Fe^2+^ were determined at 5, 20, 30, and 37°C in 9KM1 medium. Mesurements were carried out in adapted cultures to the corresponding temperature from an initial inoculum at 20°C. As seen in Figures 3A,B, PG05 and MC2.2 strains showed an increase in the cellular density in function of time between 20 and 37°C, being 20°C their optimum temperature. At optimum temperature, the exponential phase for both strains was between days 1 and 7. For the PG05 strain, the maximum cell density obtained was 1.031 × 10^9^ cells/mL, with a growth rate of 0.0493 h^–1^ and a doubling time of 14.06 h. In comparison, the MC2.2 strain reached a maximum cell density of 1.131 × 10^9^ cells/mL, with a growth rate of 0.054 h^–1^, and a doubling time of 12.83 h. Additionally, when the strains PG05 and MC2.2 were cultured at 5°C, they grew marginally, reaching 1.698 × 10^8^ and 1.950 × 10^8^ cells/mL with doubling times of 24.73 and 44.29 h, respectively. As shown in Figure 3C, the doubling reached a minimum at 20°C in both PG05 and MC2.2 strains. In addition, in each strain the doubling time estimated at 20°C was significantly lower than those estimated at 5 and 30°C (*P* < 0.05). The doubling times values did not show significant differences between strains at 5 and 20°C, but a slightly more negative effect was observed for the strain PG05 at 30 y 37°C. Considering the growth profile at different temperatures, both PG05 and MC2.2 strains were classified as psychrotolerant (Kupka et al., 2007). Interestingly, as expected, these strains showed a significantly higher growth rate at 20°C than the mesophilic type strain ATCC 23270 (data not shown).

In previous studies, the culturing of acidophilic iron-oxidizing microorganisms from low temperature areas has led to the isolation of several strains of *Acidithiobacillus ferrivorans* (Barahona et al., 2014; González et al., 2014; Peng et al., 2017). They are mainly characterized by their ability to oxidize Fe^2+^ with more efficiency than *At. ferrooxidans* at 5°C (Kupka et al., 2007; Escobar et al., 2010; Hallberg et al., 2010). In agreement, *At. ferrivorans* presents higher growth rates at 5°C (*t*_*d*_ > 50 h) in standard 9K medium (33.3% Fe^2+^). In *At. ferrivorans* SS3, the generation time at 5°C was 43–58 h (Kupka et al., 2007), while an isolate of *At. ferrooxidans* grown at 2 and 6 °C showed a generation time of 247–103 h, respectively (Ferroni et al., 1986). In addition, the strain NO-37 of *A. ferrivorans* can grow optimally at 30°C, reaching a doubling time of 3.2 h (Hallberg et al., 2010). In our experimental setting, at 5 °C the strains PG05 and MC2.2 attained doubling times of 25 and 44 h. Thus, these strains seem to be better adapted for growing at low temperatures than others strains from the same species, and they have performances similar to those observed of other psychrotolerant *At. ferrivorans*.

### Phylogenomic and pangenomic characterization of PG05 and MC2.2 strains

#### General genomic features

The assembled genome of PG05 strain consisted of 2,764,394 bp, 288 contigs, and 2,963 coding sequences with an average G+C content of 58.6 % (Table 4). On the other hand, the MC2.2 strain possesses a genome of 2,838,093 bp, 328 contigs, and 3,118 coding sequences, with an average G+C content of 59 %. The G+C content values are similar to those of *At. ferrooxidans* (averaged 58.49% in eleven strains, in Supplementary Table 2 (Supporting information)], and above the 56.5% reported for *At. ferrivorans* (Hallberg et al., 2010).

**TABLE 4:** Genome sequence attributes of *Acidithiobacillus* strains PG05 and MC2.2.

	Strain	
	
Characteristic	PG05	MC2.2
Organism	*Acidithiobacillus ferrooxidans*	*Acidithiobacilus ferrooxidans*
Place of Isolation	Puerto Guadal, Chilean Patagonia	Marian Cove, King George Island, Antarctic
Culture number	DSM 102806	JCM5614
Genome accession	JAEQBE000000000	JAEQBG000000000
GenBank assembly accession	GCA_017165965.1	GCA_017165975.1
BioProject ID	PRJNA689776	PRJNA689776
Biosample	SAMN17216812	SAMN17216810
Contigs	288	328
Genome Size (bp)	2,764,394	2,838,093
G + C content (%)	58.6	59
N50	19,061	18,793
Genome Coverage	57.29X	57.29X
Repeats region	1	1
CDS total	2,963	3,118
CDS coding	2,877	2,978
Genes (RNA)	51	58
Pseudo genes	86	82
rRNAs (5S, 16S, 23S)	1, 1, 1	1, 4, 3
tRNAs	44	46
ncRNAs	4	4
Plasmids	0	0

#### Phylogenomic and pangenomic analysis

**FIGURE 4:**
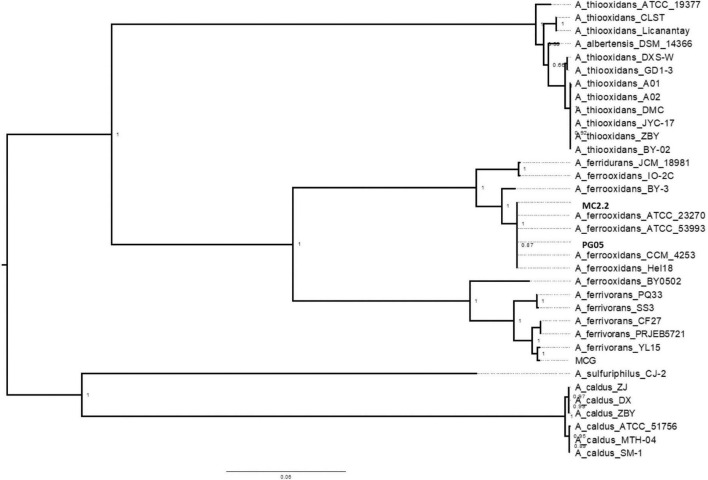
Dendrogram constructed by the maximum-likehood method showing the phylogenetic relationships using 31 gene sequences of bacterial species of the genus *Acidithiobacillus*. Bootstrap values expressed as percentages of 1,000 replications are shown. Bars represent 1 substitution per 100 nucleotides.

**FIGURE 5:**
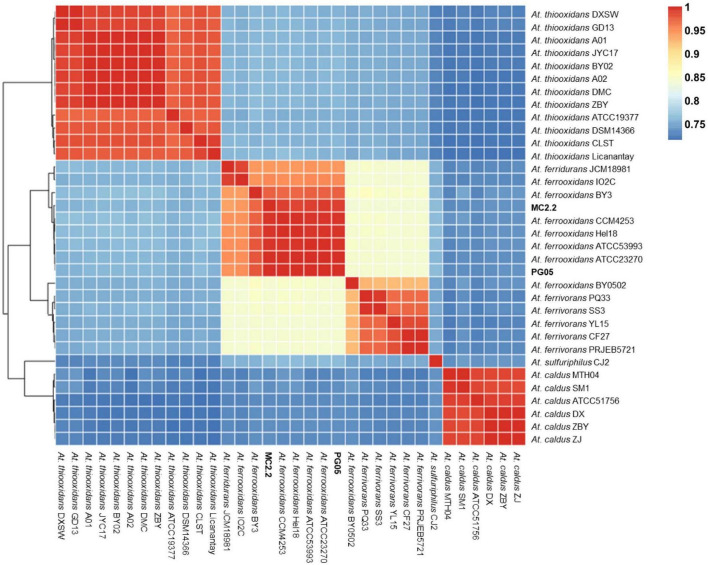
Whole genome nucleotide identity and multi-locus phylogenetic analysis. Average nucleotide identity (ANI) in the 32-genome *Acidithiobacillus* dataset.

In order to assess the relationships between newly sequenced PG05 and MC2.2 strains and other known representatives of the *Acidithiobacillus* genus, the genome sequences were compared by performing a multi-locus (with thirty marker genes) phylogenetic analysis and estimation of average nucleotide identity (ANI) as described in Materials and Methods. As shown in Figure 4, phylogenetic analysis demonstrated that both strains are related to *At. ferrooxidans*, since they are located in the same clade as the strains ATCC 23270, ATCC 53993, CCM 4253 and Hel18 of this species. In addition, the genomes of the strains PG05 and MC2.2 exhibited an ANI of 99.9 and 99.2% when compared to the genome of *At. ferrooxidans* ATCC 23270, respectively, while the ANI values compared to *At. ferrivorans* were substantially lower in both cases (84%; Figure 5). The values are in agreement with those previously reported for other strains of these species (Peng et al., 2017). With these results, we conclude that both strains are phylogenetically affiliated with *At. ferrooxidans* species, so hereinafter these strains will be referred to as *At. ferrooxidans* PG05 and MC2.2.

In order to provide a genomic overview of the species and to highlight the unique genomic characteristics of the strains of *At. ferrooxidans* PG05 and MC2.2, a pangenome analysis was undertaken using the available genome sequences of nine *At. ferrooxidans* strains. Orthologue detection allowed the identification of the core, accessory, and singleton genomes. The characteristics of the genomes of the nine *At. ferrooxidans* strains available in the NCBI database are given in Supplementary Table 2 (Supporting information). A list of genes, including pangenome subdivision and clusters of orthologous groups of proteins (COGs), for each strain, is available in Supplementary Table 3 (Supporting information).

**FIGURE 6:**
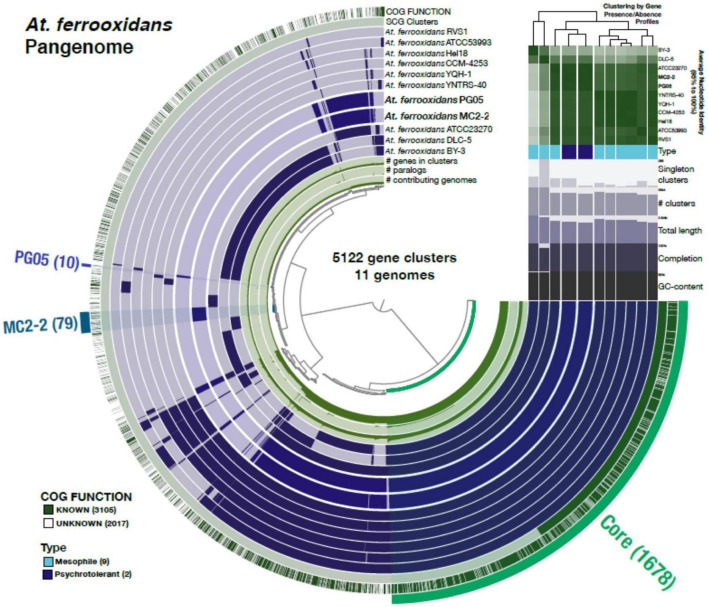
The *At. ferrooxidans* pangenome. Each one of the 5,122 gene clusters contains one or more genes contributed by one or more isolate genome. The middle 11 layers are the genomes and the bars indicate the occurrence of a given gene cluster in that strain. The top two outside layers describe (1) the gene clusters in which at least one gene was functionally annotated using COGs and (2) the single copy core-gene clusters across the 11 genomes. The bottom three inside layers show (1) the number of genes in the corresponding cluster; (2) the number of paralogs in the corresponding cluster and (3) the number of genomes on which the corresponding cluster is present. Finally, the outer selections correspond to the Core gene clusters and those exclusively present (“Singletons”) in PG05 and MC2.2. The Average Nucleotide Identity values among the genomes from the high (green) to low (white) similarity are also shown, and the dendrogram on the top represents the hierarchical clustering of genomes based on the occurrence of gene clusters.

**FIGURE 7:**
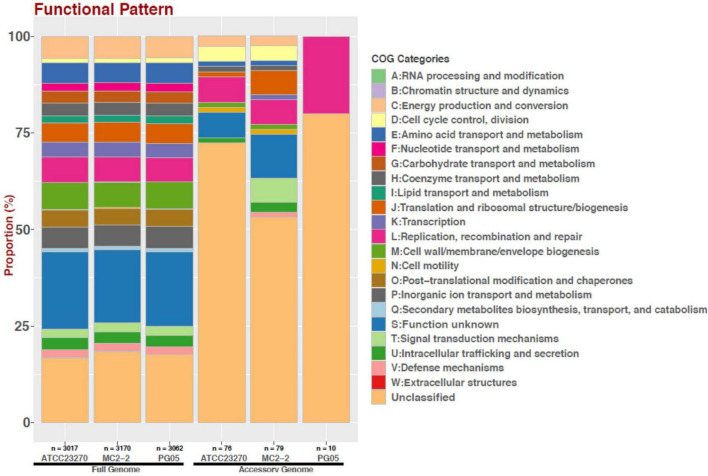
Distribution of COG functional categories for full and accessory genomes of strains ATCC 23270, MC2.2 and PG05 of *At. ferrooxidans*.

As shown in Figure 6, the total gene clusters from the genomes were calculated in 5122 CDSs (pangenome), and 1,678 genes were shared by the eleven genomes surveyed (core genome). Scrutinizing each genome, 10 and 79 unique genes were identified in PG05 and MC2.2 genomes, respectively, while we identified 76 unique genes in *At. ferrooxidans* ATCC 23270. The accessory genome also showed important differences in the composition of the COG category (Figure 7). The strain MC2.2 showed a higher content of accessory genes than PG05 and ATCC 23270, and the genes were related to translation and ribosome structure and biogenesis, signal transduction, intracellular trafficking and secretion, and defense mechanisms. In the PG05 strain, the accessory genome was dominated by genes of the replication, recombination, and DNA repair COG category. An important percentage of genes for unclassified and unknown proteins were detected in accessory genomes of both PG05 (80%) and MC2.2 (53%) strains.

### Stress resistance and defense mechanisms

#### Low temperatures

In cold areas like Patagonia and Antarctic, low temperature can represent an important selective pressure on microorganisms. Studies have demonstrated that temperature has significant effects on microbial community structure (Chen et al., 2014; Wang et al., 2018) and bioleaching efficiency for different metals (Vakylabad, 2011; Xiao et al., 2017). *At. ferrivorans*, the most prominent known psychrotolerant acidophilic bacterium, is able to tolerate low temperature by mechanisms that involve synthesis and accumulation of compatible solutes (trehalose), membranes with desaturated fatty acids, and the activity of cold-shock proteins, among other mechanisms (Dopson et al., 2007; Liljeqvist et al., 2012; Christel et al., 2016). In this work, a search for genes encoding for these functions revealed that both PG05 and MC2.2 strains have a significantly higher content of genes for cold tolerance (21 and 29, respectively) than the type strain ATCC 23270 (14). In both cases, genes appear related to trehalose biosynthesis, Dead/Deah-box ATP-dependent RNA helicase activity, fatty acid desaturase, glycine betaine transport, and hopanoid biosynthesis associated with RND transporters. It is remarkable that the Antarctic strain MC2.2 possesses genetic redundancy for some of these genes; specifically, it contains 3 genes for cold shock protein CspE and 2 genes for fatty acid desaturase, while PG05 and ATCC 23270 possess only one gene for each of these functions. It is noticeable that redundancy of genes related to cold tolerance has also been reported in strains of the cold-adapted bacterium *At. ferrivorans* (Ccorahua-Santo et al., 2017). These data clearly show that both strains possess the genetic potential for adaptation to low temperatures. However, as expected, the Antarctic strain is better equipped than the Patagonian strain, and both are significantly better equipped than the type strain.

#### Oxidative stress

In the extremely acidic conditions found in natural acid rock drainages, microorganisms have to deal with an abundant supply of metals. Iron is required as a micronutrient by all acidophiles, and as a primary energy source for iron oxidizers. However, iron and other metals like copper, cadmium, zinc and cobalt can also induce oxidative damage to biomolecules by generating reactive oxygen species (ROS). Therefore, acidophilic microorganisms are often exposed to highly oxidizing conditions and face the problem of maintaining intracellular redox homeostasis (Ferrer et al., 2016a). Enzymatic and non-enzymatic antioxidants mechanisms involved in protection against oxidative stress (Imlay, 2019) are thus deemed essential for adaptation and survival in these environments. A variety of these antioxidant systems have been described in *Acidithiobacillus* and in other iron oxidizers (Ferrer et al., 2016b; Bellenberg et al., 2019; Feng et al., 2020; González et al., 2020).

Our analysis revealed that genomes of *At. ferrooxidans* PG05 and MC2.2 harbor a plethora of genes for proteins involved in oxidative defense including ROS scavenging SOD, thiolperoxidase, alky hydroperoxidereductase (AhpC), peroxiredoxin and catalase, and systems based on glutaredoxin and thioredoxin for thiol/disulfide redox homeostasis (Table 5). The strain MC2.2 carries a higher content and redundancy of genes including, for example, 3 genes for glutaredoxin and AhpC, and 5 genes for SOD (Fe/Mn-SOD). It is noteworthy that this strain harbors a gene for catalase, an enzyme that is characteristic of aerobic pathogenic bacteria (Zamocky et al., 2008), but is underrepresented in acidophiles (Cárdenas et al., 2012), and so far has not been described in members of the genus *Acidithiobacillus*. This enzyme does not appear to be present in the PG05 strain or in the type strain.

**TABLE 5:** Genes associated with cold resistance and oxidative stress identified in the genomes of strains PG05 and MC2.2 compared to the type strain ATCC 23270.

Gene	Product	Acidithiobacillus ferrooxidans		
		
		PG05	MC2.2	ATCC 23270
**Cold resistance genes**				
treT	Alpha-Trehalose synthase	1	2	1
treY	Malto-oligosyltrehalose synthase	5	4	1
treZ	Malto-oligosyltrehalose trehalohydrolase	2	3	1
susA	Sucrose synthase	1	1	1
Csp	Cold shock protein	1	3	1
cshA	DEAD/DEAH box ATP-dependent RNA helicase DeaD	3	4	3
Tig	Trigger factor	1	4	1
desA	Fatty acid desaturase	1	2	1
hpnN	Hopanoid biosynthesis associated RND transporter	2	2	0
hpnH	Hopanoid biosynthesis associated radical SAM protein	2	1	2
proP	Glycine betaine transport	2	3	2
	**Total**	**21**	**29**	**14**
**Oxidative stress response genes**				
Grx	Glutaredoxin	2	3	2
Ahp	Alkyl hydroperoxidereductase	2	3	1
Sod	Superoxide dismutase	1	5	1
Prx	Peroxiredoxin	2	3	3
Cat	Catalase	0	1	0
tpx	Thiol peroxidase	1	3	1
trxA	Thioredoxin	3	3	6
trxB	Thioredoxin reductase	1	2	1
gshB	Glutathione synthetase	1	1	2
	**Total**	**13**	**24**	**17**

**TABLE 6:** Genes associated with metal tolerance identified in the PG05 and MC2.2 strain genomes compared to the type strain ATCC 23270.

Metal	Gene	Product	*Acidithiobacillus ferrooxidans*		
			
			PG05	MC2.2	ATCC 23270
Copper	*copA*	Copper-translocating P-type ATPase CopA	1	1	1
	*copB*	Copper-translocating P-type ATPase CopB	1	1	1
	*copC*	Copper resistance protein CopC	1	1	1
	*copD*	Copper resistance protein CopD	1	1	1
	*cop_like*	Copper-translocating P-type ATPase putative	1	1	1
	*cusA*	Cation efflux system protein CusA	1	1	1
	*cusB*	Cation efflux system protein CusB	1	1	1
	*cusC*	Cation efflux system protein CusC	1	1	1
	*cusF*	Copper binding protein CusF	2	2	2
	*cusS*	Heavy metal sensor histidine kinase	1	1	1
	*cusR*	Heavy metal response regulator transcription factor	1	1	1
	*ppk*	Polyphosphate:ADP/GDP phosphotransferase	2	2	2
	*ppx*	Exopolyphosphatase	1	1	1
	*copZ*	Copper(I) chaperone	2	2	2
	*AFE_0022*	Cation channel protein, putative	1	1	1
	*AFE_0326(CusF)*	Cation efflux system protein, putative	1	1	1
	*AFE_1818*	Plasma-membrane proton-efflux P-type ATPase	1	1	1
	*AFE_0329*	Copper-translocating P-type ATPase	1	1	1
	*AFE_1143*	Heavy metal efflux transporter, MFP subunit, putative	0	0	1
	*znuA_like*	Zinc ABC transporter substrate-binding protein	0	0	0
	*cadA*	Cadmium-transporting ATPase	1	1	1
	*nlpE*	Copper resistance protein	1	1	0
Zinc	*znuA*	Zinc ABC transporter, substrate-binding protein ZnuA	1	1	1
	*znuC*	Zinc ABC transporter, ATP-binding protein ZnuC	1	1	1
	*znuB*	Zinc ABC transporter, permease protein ZnuB	2	2	2
	*czcA*	CzcABC family efflux RND transporter, transmembrane protein	4	4	4
	czcB	RND efflux system, membrane fusion protein	3	3	3
	*czcC*	CzcABC family efflux RND transporter, outer membrane protein	4	4	4
	*czcD*	Cobalt/zinc/cadmium resistance protein CzcD	3	3	3
	*AFE_1788*	Membrane-associated zinc metalloprotease, putative	1	1	1
Lead	*cmtR*	Cd(II)/Pb(II)-responsive transcriptional regulator, CmtR	1	1	1
Mercury	*merA*	Mercury reductase	2*	4*	1
	*merB*	Organomercurial lyase	0	0	0
	*merC*	Inner membrane protein	1	2*	1
	*merD*	Transcriptional regulator	2*	2*	0
	*merE*	Mercuric resistance protein	1	2*	1
	*merP*	Mercury binding protein	0	3*	0
	*merR*	Hg(II)-responsive transcriptional regulator	0	2*	0
	*merT*	Mercuric ion transport protein	0	5*	0
	*merR-like*	MerR family transcriptional regulator	4	5*	4
		Total	**53**	**68**	**50**

*There are one or more incomplete genes identified in NCBI or SnapGene programs with different percentage identities between them.

Peroxidases are considered the primary scavengers when the dose of H_2_O_2_ is in the low-micromolar range, the most-common range found in nature. Usually, it is assumed that catalase activity predominates at higher doses, when peroxidases are saturated due to the slowness of electron delivery and/or inactivation by over-oxidation (Mishra and Imlay, 2012). Thus, it is possible to predict that the MC2.2 strain could be exposed to higher levels of H_2_O_2_. Obviously, low temperatures, and likely high UV irradiation prevailing in the Antarctic could represent selective forces in favor of fixing genes involved in antioxidative protection. Cold can directly induce oxidative stress by increasing the concentration of dissolved oxygen and thus favoring the generation of ROS (Das and Roychoudhury, 2014). Induction of redox stress by low temperature exposure has been previously established for other microorganisms (Dahlsten et al., 2014; Kumar et al., 2020).

#### Metal tolerance

It is a well-known fact that in acidic environments, a high concentration of dissolved metal cations like iron, copper, zinc, cobalt, cadmium, uranium among others can be found (Navarro et al., 2013; Ferrer et al., 2016a). The positive charge of the cell surface of acidophiles has the effect of helping to keep metal cations out of the cytoplasm (Falagan and Johnson, 2018). However, an additional number of specialized systems have been described in several acidophiles as means of tolerating metals. These mechanisms include a number of membrane protein families involved in metal extrusion (Almárcegui et al., 2014; Martínez-Bussenius et al., 2016), as well as mechanisms involved in complexation and precipitation of metals (Jia et al., 2010; Dopson and Holmes, 2014). Additionally, as mentioned above, a key role of intracellular deposits of polyP has been described (Orell et al., 2012; Panyushkina et al., 2019). In this study, the predicted mechanisms include a number of transporters for copper, zinc and lead, most of which could be detected in both PG05 and MC2.2 strains. In these strains, metal efflux could occur through transport systems composed of the ATP-binding cassette (ABC) superfamily and major facilitator superfamily (MFS) proteins, as well as cation translocating P-type ATPases (Table 5 and Supplementary Table 2). CzcABC is a representative of the RND-type efflux system and confers resistance to cobalt, zinc and cadmium (Klonowska et al., 2020). The occurrence of multiple copies of *czcC* (n = 4) in both strains could confer more activity and/or versatility to the system for the translocation of several heavy metals. Interestingly, we also detected genes involved in the biosynthesis (*ppk*) and degradation (*ppx*) of polyP granules; the *ppx* gene that encodes exopolyphosphatase was duplicated in each strain. This enzyme is responsible for the degradative activity of polyP by hydrolyzing terminal residues of polyP and releasing Pi (Kornberg, 1999). PolyP can substitute ATP in kinase reactions, thus having a role in energy supply. Furthermore, these macromolecules serve as a chelator of metals, thus contributing to metal tolerance. PolyP has been described as having a role in copper tolerance in *At. ferrooxidans*, *At. caldus*, *At. ferrivorans*, and *Sulfobacillus thermotolerans*, among others (Navarro et al., 2013; Panyushkina et al., 2019). In addition, polyP plays a role in the physiological adjustments of bacteria to environmental changes and stress conditions and they have been described as conferring tolerance to heat, oxidants, osmotic challenge, acid, and UV radiation, among other factors (Crooke et al., 1994; Rao and Kornberg, 1996; Tsutsumi et al., 2000; Kim et al., 2002). Thus, the presence of polyP intracellular deposits may be a mechanism favoring adaptation to a variety of environmental challenges in these microorganisms.

Interestingly, the MC2.2 strain has an abundant number of mercury resistance genes that are scattered throughout the genome. In bacteria, the *mer* operon (*merRTPCDAB*) confers mercury resistance. These genes are often located in plasmids or transposons, but may also be found in chromosomes. MerP scavenges and transports inorganic Hg^2+^ to MerT at the plasma membrane, and then MerT transports it inside the cells where it is reduced to Hg^0^ by the flavoenzyme mercury reductase MerA (Barkay et al., 2003; Zhang et al., 2020). MerE is another Hg^2+^ transporter, while MerF and MerC mediate phenylmercury transport into cells (Sone et al., 2013). MerR is an activator/repressor that regulates the expression of the structural genes of the operon. In a few mercury resistance operons, a second regulator gene, *merD*, is present and binds weakly to the MerR operator site to down-regulate the system (Ravel et al., 2000). In the genomic analysis, a putative operon containing the *merCAD* genes was detected, in addition to three operons containing the *merTP* genes, and two operons containing the *merT/merR* genes were also found. Furthermore, it was possible to detect two non-identical partial copies of the *merA* gene, and one copy of the *merD* and *merE* genes, respectively. In this way, the analysis revealed a high diversity and redundancy of *mer* genes, suggesting the presence of a variety of mercury resistance mechanisms. The content of *mer* genes in the MC2.2 strain differs clearly from the PG05 and the ATCC 232270 type strain, which, although they have a putative operon containing the *merCAD* genes, do not have *merT/merP* or other genes for resistance.

Mercury is naturally present in the Antarctic environments. However, its deposition increases during the Antarctic spring, when the returning sunlight causes the melting of sea ice and release of mercury that falls onto sea ice and the ocean. Thus, the microorganisms that inhabit Antarctic environments such as snow, brine, frost flowers and recently-formed sea ice, possess the Hg detoxifying *me*r gene (Gionfriddo et al., 2016). Even so, the presence of an abundant dosage of *mer* genes in the Antarctic strain MC2.2 is a novel finding for iron-oxidizing representatives of the *Acidithiobacillus* genus.

## Conclusion

The Patagonian PG05 and Antarctic MC2.2 strains were found to be psychrotolerant with optimal temperatures of 20°C. Phylogenetic and phylogenomic analyzes indicated that both PG05 and MC2.2 strains correspond to *At. ferrooxidans*. In the PG05 strain, internal polyphosphate deposits could contribute to its adaptation to environments with a high content of dissolved metals and other extreme factors. The pangenomic analysis of PG05 and MC2.2 genomes showed the presence of 10 and 79 unique genes, respectively, with differences in the composition of the COG category. An important percentage of genes for unclassified and unknown proteins were detected in accessory genomes of these strains. The Antarctic strain MC2.2 showed a high dose of genes for resistance to mercury and adaptation to low temperatures. This work constitutes the first report of strains adapted to the cold of *At. ferrooxidans*, and opens new perspectives to meet the current challenges of the metal bioleaching industry.

## Data availability statement

The original contributions presented in this study are included in the article/Supplementary material, further inquiries can be directed to the corresponding author.

## Author contributions

GL and BD: design of the project. GL: supervision, project administration, and funding acquisition. GL, CM-V, JG-G, JA-A, and AF: methodology, formal analysis, and investigation. CM-V, AF, and XV: sample collection. GL and CM-V: writing original draft and conceptualization. All authors contributed to the article and approved the submitted version.

## Footnotes

1
https://www.ncbi.nlm.nih.gov/

2
https://www.ncbi.nlm.nih.gov/genome/annotation_prok/

3
https://github.com/microgenomics/plotMyGBK

4
https://github.com/Rdatatable/data.table

5
http://tree.bio.ed.ac.uk/software/figtree/
