# Supplementary material for: Characterization and genomic analysis of two novel psychrotolerant Acidithiobacillus ferrooxidans strains from polar and subpolar environments
Source: Front Microbiol. 2022 Aug 24;13:960324. doi: 10.3389/fmicb.2022.960324 (PMC9449456; doi:10.3389/fmicb.2022.960324)
Supplement: Supplementary file 2 [file Table_1.DOCX]

**Table S1**. GenBank accession number of *Acidithiobacillus*´s genome sequences used for phylogenetic analysis in this study.

| **N°** | **Strain** | **Genome Accession Number** |
| --- | --- | --- |
| 1 | *At. thiooxidans* ATTC 19377 | NZ_SZUV00000000.1 |
| 2 | *At. thiooxidans* CLST | NZ_LGYM00000000.1 |
| 3 | *At. thiooxidans* Licanantay DSM17318 | NZ_JMEB00000000.1 |
| 4 | *A. albertensis* DSM 14366 | NZ_MOAD00000000.1 |
| 5 | *At. thiooxidans* DXS-W | NZ_LWRY00000000.1 |
| 6 | *At. thiooxidans* GD1-3 | NZ_LWSC00000000.1 |
| 7 | *At. thiooxidans* A01 | NZ_AZMO00000000.1 |
| 8 | *At. thiooxidans* A02 | NZ_LWSA00000000.1 |
| 9 | *At. thiooxidans* DMC | NZ_LWSB00000000.1 |
| 10 | *At. thiooxidans* ZBY | NZ_LZYI00000000.1 |
| 11 | *At. thiooxidans* BY-02 | NZ_LWRZ00000000.1 |
| 12 | *At. ferridurans* JCM 18981 | NZ_AP018795.1 |
| 13 | *At. ferrooxidans* IO-2C | NZ_PQJK00000000.1 |
| 14 | *At.* *ferrooxidans* BY-3 | NZ_AZNR00000000.1 |
| 15 | *At. ferrooxidans* ATCC 23270 | NC_011761.1 |
| 16 | *At*. *ferrooxidans* ATCC 53993 | NC_011206.1 |
| 17 | *At. ferrooxidans* CCM 4253 | NZ_QKQP00000000.1 |
| 18 | *At. ferrooxidans* Hel18 | NZ_LQRJ00000000.1 |
| 19 | *At. ferrooxidans* BY0502 | NZ_LVXZ00000000.1 |
| 20 | *At. ferrivorans* PQ33 | NZ_LVZL00000000.1 |
| 21 | *At. ferrivorans* SS3 | NC_015942.1 |
| 22 | *At. ferrivorans* CF27 | NZ_CCCS000000000.2 |
| 23 | *At. ferrivorans* PRJEB5721 | NZ_LT841305.1 |
| 24 | *At. ferrivorans* YL15 | NZ_MASQ00000000.1 |
| 25 | *At. sulfuriphilus* CJ-2 | NZ_RIZI00000000.1 |
| 26 | *At. caldus* ZJ | NZ_LZYG00000000.1 |
| 27 | *At. caldus* DX | NZ_LZYE00000000.1 |
| 28 | *At. caldus* ZBY | NZ_LZYF00000000.1 |
| 29 | *At. caldus* ATCC 51756 | NZ_CP005986.1 |
| 30 | *At. caldus* MHT-04 | NZ_CP026328.2 |
| 31 | *At. caldus* SM-1 | NC_015850.1 |
